# Challenges in the Analysis of Longitudinal Pain Data: Practical Lessons from a Randomized Trial of Annular Closure in Lumbar Disc Surgery

**DOI:** 10.1155/2019/3498603

**Published:** 2019-02-03

**Authors:** Gerrit J. Bouma, Martin Barth, Larry E. Miller, Sandro Eustacchio, Charlotte Flüh, Richard Bostelmann, Senol Jadik

**Affiliations:** ^1^Department of Neurosurgery, OLVG and Amsterdam University Medical Centers, Amsterdam, Netherlands; ^2^Department of Neurosurgery, Klinikum Frankfurt Höchst, Frankfurt, Germany; ^3^Miller Scientific Consulting, Inc., Asheville, NC, USA; ^4^Department of Neurosurgery, Medical University Graz, Graz, Austria; ^5^Department of Neurosurgery, University Medical Center Schleswig-Holstein, Kiel, Germany; ^6^Department of Neurosurgery, University Hospital Düsseldorf, Düsseldorf, Germany

## Abstract

*Purpose. *To analyze leg pain severity data from a randomized controlled trial (RCT) of lumbar disc surgery using integrated approaches that adjust pain scores collected at scheduled follow-up visits for confounding clinical events occurring between visits.* Methods.* Data were derived from an RCT of a bone-anchored annular closure device (ACD) following lumbar discectomy versus lumbar discectomy alone (Control) in patients with large postsurgical annular defects. Leg pain was recorded on a 0 to 100 scale at 6 weeks, 3 months, 6 months, 1 year, and 2 years of follow-up. Patients with pain reduction ≥20 points relative to baseline were considered responders. Unadjusted analyses utilized pain scores reported at follow-up visits. Since symptomatic reherniation signifies clinical failure of lumbar discectomy, integrated analyses adjusted pain scores following a symptomatic reherniation by baseline observation carried forward for continuous data or classification as nonresponders for categorical data.* Results.* Among 550 patients (272 ACD, 278 Control), symptomatic reherniation occurred in 10.3% of ACD patients and in 21.9% of controls (*p *< 0.001) through 2 years. There was no difference in leg pain scores at the 2-year visit between ACD and controls (12 versus 14;* p* = 0.33) in unadjusted analyses, but statistically significant differences favoring ACD (19 versus 29;* p* < 0.001) in integrated analyses. Unadjusted nonresponder rates were 6.0% with ACD and 6.7% with controls (*p* = 0.89), but 15.7% and 27.8% (*p* = 0.001) in integrated analyses. The probability of nonresponse was 16.4% with ACD and 18.3% with controls (*p* = 0.51) in unadjusted analysis, and 23.7% and 31.2% (*p* = 0.04) in integrated analyses.* Conclusion*. In an RCT of lumbar disc surgery, an integrated analysis of pain severity that adjusted for the confounding effects of clinical failures occurring between follow-up visits resulted in different conclusions compared to an unadjusted analysis of pain scores reported at follow-up visits only.

## 1. Introduction

Long-term follow-up in clinical trials is mandatory for monitoring the safety and efficacy of chronic pain treatments. However, longer follow-up periods also increase the likelihood for missing data, patient withdrawal, and clinical events that may confound data interpretation. Numerous analytic approaches are available to deal with missing data (e.g., multiple imputation, last observation carried forward, baseline observation carried forward [BOCF], and nonresponder imputation) and their comparative performance and recommendations for use are well-documented [1–3]. However, methods to adjust existing data for confounding clinical events have received considerably less attention.

Take, for example, a clinical trial of lumbar disc surgery with annual follow-up visits for 5 years. A patient reports pain severity of 20 on a 100 mm visual analogue scale (VAS) at 1-year follow-up, undergoes repeat discectomy at 1.5 years due to increasing pain and disability from reherniation, and reports back pain severity of 20 at the scheduled 2-year follow-up visit (6 months after reoperation). In this instance, how should pain scores be treated in the analysis? Excluding data from patients who undergo reoperation is an inherently biased method of analysis. Analysis of pain scores reported only at the annual follow-up intervals fails to account for the implicit episode of increased pain and eventual clinical failure (i.e., reoperation) occurring during the interim period. Further, the 2-year pain scores reported in this example are mainly attributable to the reoperation, not to the index surgery. Thus, in clinical trials of pain treatments with planned long-term follow-up, confounding clinical events occurring during the interval between scheduled visits should be taken into account in data analysis. The purpose of this study was to analyze leg pain severity data from a clinical trial of lumbar disc surgery using integrated approaches that adjusted pain scores collected at scheduled follow-up visits for confounding clinical events that occurred between visits.

## 2. Methods

### 2.1. Data Source

Data were derived from a multicenter randomized controlled trial intended to determine whether implantation of a bone-anchored annular closure device (ACD) in patients with a large annular defect following lumbar discectomy reduced the risk of recurrent herniation compared to lumbar discectomy alone (controls). The clinical trial was approved by the local ethics review boards, and all participants provided written informed consent. This study was prospectively registered at ClinicalTrials.gov (NCT01283438). The study design [4] and 2-year results [5] of this trial have been described elsewhere. Briefly, eligible patients were 21 to 75 years of age, with single-level lumbar disc herniation, with disc height at least 5 mm, and who were unresponsive to at least 6 weeks of nonsurgical treatment. All patients had lumbar radiculopathy with positive straight leg raise or femoral stretch test, Oswestry Disability Index score (ODI) of at least 40 (0-100 scale), and leg pain severity of at least 40 on a 100 mm VAS. Patients were randomized intraoperatively following verification of a large annular defect (height 4-6 mm; width 6-10 mm), which is a known risk factor for reherniation and reoperation [6].

### 2.2. Outcomes

Leg pain severity in postsurgical follow-up was recorded on a 100 mm VAS scale at scheduled visits (6 weeks, 3 months, 6 months, 1 year, and 2 years) using a 1-week recall period. Importantly, pain experienced by patients in the period between follow-up visits was not quantitatively recorded. Patients reporting pain reduction of at least 20 points relative to baseline were considered responders. Symptomatic reherniation was defined as a recurrent herniation that was surgically verified during reoperation, reported as an adverse event, or identified by the imaging core laboratory in conjunction with the patient reporting moderate disability (ODI ≥ 40), radicular symptoms, or neurologic deterioration.

### 2.3. Statistical Analysis

A modified intent-to-treat population was analyzed that included all randomized patients in whom the intended procedure was attempted. We analyzed leg pain data in four different ways, with two methods utilizing continuous data (leg pain VAS) and two methods utilizing categorical data (responder versus nonresponder). For each method, we drew comparisons between unadjusted and integrated results. Unadjusted analyses of group means and nonresponse rates utilized pain scores reported at scheduled follow-up visits. Integrated analyses considered patients with symptomatic reherniation as clinical failures. For integrated analyses of continuous data, we used a BOCF approach where the baseline observation was carried forward to adjust pain scores at all subsequent follow-up visits. This method was selected since symptomatic reherniation signifies clinical failure of lumbar discectomy and because VAS pain scores at the time of reherniation are comparable to the baseline VAS scores attributable to primary herniation in the current study [7]. For integrated analyses of categorical data, patients with symptomatic reherniation were classified as nonresponders at all subsequent follow-up visits, again to signify clinical failure of the primary lumbar discectomy procedure.

Group comparisons of mean leg pain scores at 2 years were performed using an independent-samples t-test. We additionally performed a repeated-measures linear mixed model, which is more robust since it considers all available leg pain scores that were reported after randomization and can flexibly include patients with missing data. The proportion of nonresponders in each group at 2 years was analyzed using Fisher's exact test. Time to first nonresponse was reported as a cumulative probability using Kaplan-Meier methods, with log-rank tests for group comparisons. In all analyses, statistical significance was set at* p *< 0.05 and hypothesis testing was two-sided. Statistical analyses were performed using SAS v9.4 (SAS Institute).

## 3. Results

Between December 2010 and October 2014, 554 patients were randomly assigned to ACD (n=276) or control (n=278). The modified intention-to-treat population included 550 patients (272 ACD, 278 controls) owing to 4 patients who were randomized to ACD but in whom implantation was not attempted because of anatomical constraints. Treatment groups were well matched at baseline. The mean age of the study population was 43 years and 59% were men. Surgery was performed at L4-L5 or L5-S1 in 97% of patients. Compliance with clinical follow-up at 2 years was 91% in each group. The frequency of symptomatic reherniation was lower with ACD versus controls (10.3% versus 21.9%;* p *< 0.001) through 2 years.

**Figure 1:**
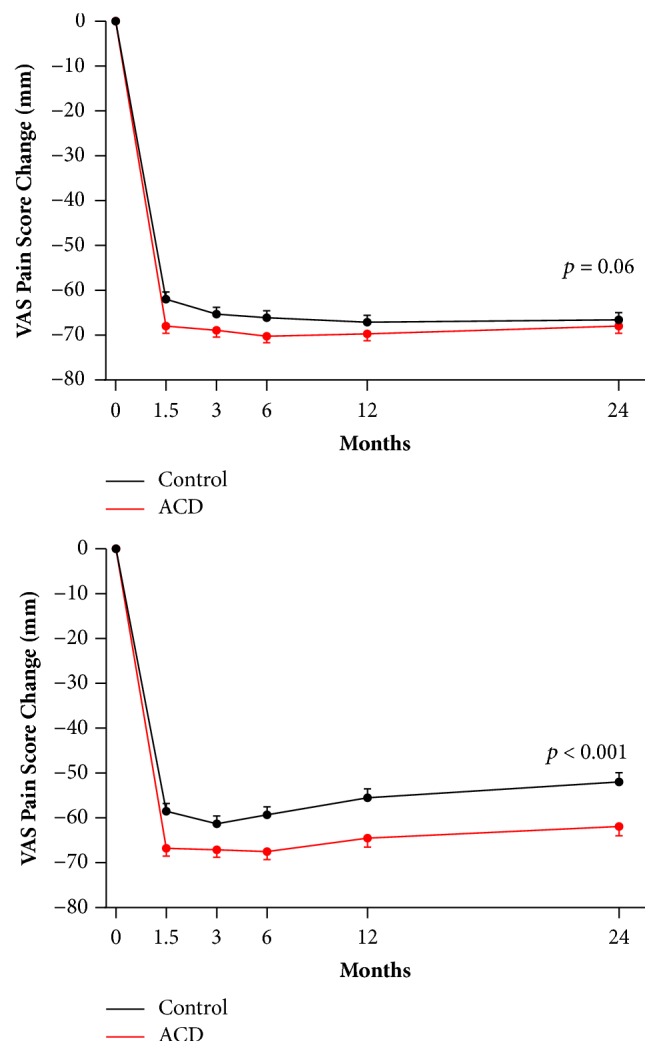
VAS pain scores with ACD and control through 2 years.** Notes:** in unadjusted analysis (top panel), values represent actual VAS scores recorded at scheduled follow-up visits. In integrated analysis (bottom panel), the BOCF approach was used where the baseline VAS pain score was substituted for the patient-reported pain score at the time of symptomatic reherniation and at each subsequent follow-up visit through 2 years. Comparing ACD to controls, VAS change from baseline (mean±SE) was -68 ± 2 versus -67 ± 2 (*p* = 0.06) in unadjusted analysis and -62 ± 2 versus -52 ± 2 (*p* < 0.001) in integrated analysis.** Abbreviations: **ACD, annular closure device; BOCF, baseline observation carried forward; SE, standard error; VAS, visual analogue scale.

In unadjusted and integrated analyses, pain scores were dramatically lower relative to baseline at each follow-up period in each group. The unadjusted analysis showed no difference in pain scores (mean ± standard deviation) between ACD and controls at the scheduled 2-year follow-up visit (12 ± 21 versus 14 ± 21;* p* = 0.33). However, the integrated analysis revealed statistically significant differences in 2-year pain scores favoring the ACD group (19 ± 30 versus 29 ± 36;* p* < 0.001) (Table 1). In the repeated measures mixed effects model, the unadjusted analysis revealed no group differences in pain score changes (mean ± standard error) between ACD and controls through 2-year follow-up (-68 ± 2 versus -67 ± 2;* p* = 0.06). However, the integrated analysis revealed statistically significant differences in pain score changes favoring the ACD group (-62 ± 2 versus -52 ± 2;* p* < 0.001) (Figure 1).

**Figure 2:**
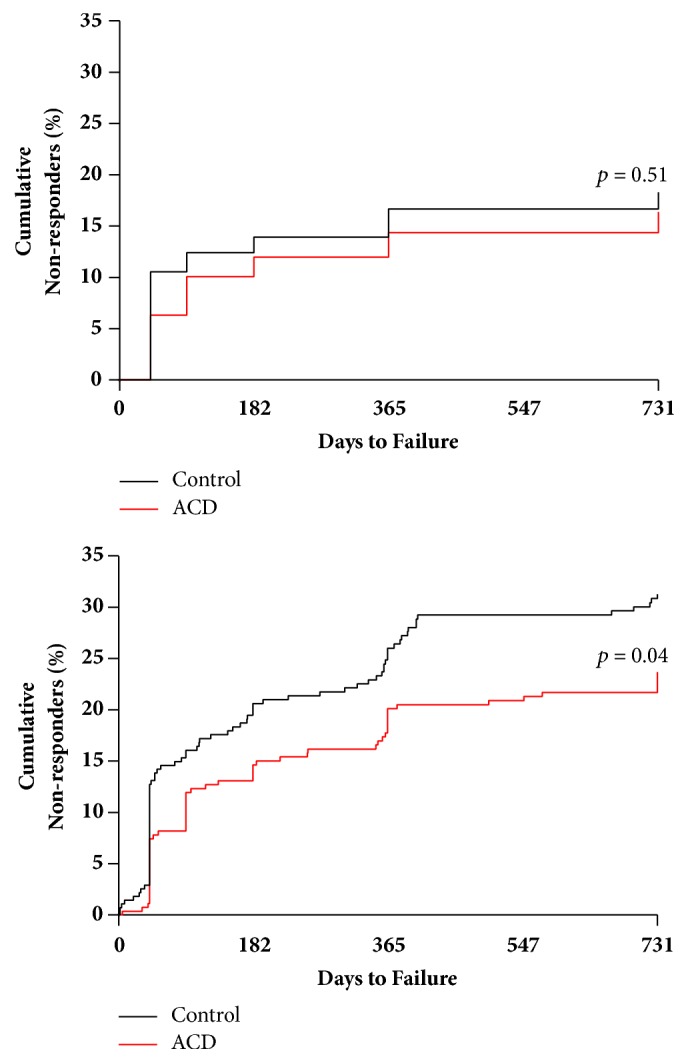
Cumulative incidence of pain nonresponders with ACD and control through 2 years.** Notes: **in unadjusted analysis (top panel), time to pain non-response defined as days to first instance of pain score improvement <20 points relative to baseline. In integrated analysis (bottom panel), time to pain nonresponse additionally includes patients at the time of symptomatic reherniation detection. Comparing ACD to controls, the cumulative incidence of pain nonresponders through 2 years was 16.4% versus 18.3% (log-rank* p* = 0.51) in unadjusted analysis and 23.7% versus 31.2% (log-rank* p* = 0.04) in integrated analysis. ACD, annular closure device; VAS, visual analogue scale.

At the scheduled 2-year follow-up visit, nonresponder rates in the unadjusted analysis were 6.0% with ACD and 6.7% with controls (*p* = 0.89). In integrated analyses, the corresponding nonresponder rates were 15.7% and 27.8%, respectively (*p* = 0.001) (Table 2). The cumulative incidence of nonresponse through 2 years was 16.4% with ACD and 18.3% with controls (*p* = 0.51) in unadjusted analysis. In integrated analysis, the corresponding cumulative nonresponse rates were 23.7% with ACD and 31.2% with controls (*p* = 0.04) (Figure 2).

## 4. Discussion

Objective and accurate measurement of pain remains a challenge in longitudinal clinical trials. Patients with degenerative spinal conditions may experience fluctuating symptoms that may not be present at scheduled follow-up visits. In this randomized controlled trial of lumbar discectomy with or without additional ACD implant, over 90% of patients in each group reported leg pain scores indicative of clinical success at 2-year follow-up, with no differences between groups. However, simply analyzing patient-reported outcomes without consideration for confounding clinical events occurring between visits will underestimate the true rate of clinical failure. In the current study, patients with symptomatic reherniation undoubtedly experienced an episode of significant pain during follow-up that was not necessarily present on the date of the scheduled follow-up visits given the 1-week recall period of the VAS pain tool. In all integrated analyses, there was a significant reduction in leg pain with ACD versus controls. These discordant results highlight the inadequacy of relying on pain scores derived solely from fixed annual follow-up intervals for clinical decision-making since most patients that experienced symptomatic reherniation during follow-up were still considered responders in the unadjusted analyses since most underwent a reoperation that biased 2-year pain scores.

An FDA recommendation for analgesic studies is development of an integrated analgesic assessment score that combines information from patient pain scores with the amount of morphine administered via patient-controlled analgesia pumps [8–10]. Other authors have echoed these sentiments by proposing development of more robust pain assessment tools that additionally consider factors such as opioid use, hospitalization days due to pain, and number of work days missed due to pain [10, 11]. The results of this study suggest that similar initiatives to develop composite markers of analgesic efficacy by combining patient-reported and clinical information may be worthwhile in clinical trials of degenerative spinal conditions.

Interestingly, the percentage of patients who required opioid analgesia at 2-year follow-up was approximately 50% lower in the ACD group versus controls. Unfortunately, these data were not collected with sufficient detail to calculate and compare equianalgesic opioid doses between groups. Nonetheless, it appears that the analysis of patient-reported pain scores may have underestimated the utility of ACD in decreasing episodic pain due to reherniation. Even the integrated analyses that accounted for clinical failures may continue to underestimate the treatment benefit since results in the ACD group were achieved with less opioid use. Future studies should implement more detailed accounting of postoperative opioid consumption, which could be used to adjust pain scores or to define a pain nonresponder if analgesia was achieved due to chronic, high-dose opioid use.

In addition to more refined analysis algorithms, more robust data collection methods could be implemented to more accurately characterize the long-term clinical course of a fluctuating condition such as sciatica. However, any initiatives to collect a greater frequency or volume of data must simultaneously avoid overburdening the patient with time-consuming questionnaires or demands for excessive on-site visits, both of which may result in the opposite of the intended effect due to patient noncompliance. Web- or phone-based questionnaires have been shown to be reliable and user-friendly and have high completion rates, which may be ideal for collecting data at more frequent intervals [12–14]. Data collection forms should be designed to record key outcome data immediately before and after significant clinical events such as reherniation, adverse events, or debilitating pain that may occur in the interim period so a patient's clinical trajectory can be tracked with greater precision instead of relying on data at routine follow-up visits only. Lastly, the common practice of withdrawing patients from a study following clinical failure should be avoided since this results in loss of statistical power and their exclusion introduces bias in the results by artificially inflating clinical response rates.

A limitation of this study is that most analyses presented here are* post hoc* in nature and should be considered exploratory. A second limitation is that imputing baseline scores at all follow-up visits following a report of symptomatic reherniation may be viewed as overly conservative. However, since all patients in this trial underwent lumbar discectomy for symptomatic lumbar disc herniation, symptomatic reherniation clearly represented failure of the primary procedure and was therefore accounted for in the analysis. The merits of this argument are obviously debatable yet seemed the most reasonable for illustrating the limitations of reporting pain scores in isolation without clinical consideration. Finally, we do not propose a single integrated analysis method for future studies, but instead present these data to highlight current limitations of pain reporting in clinical trials and to encourage additional research on this topic.

## 5. Conclusions

In a randomized controlled trial of lumbar disc surgery, an integrated analysis of pain severity that adjusts for the confounding effects of clinical failures results in different conclusions compared to an unadjusted analysis of pain scores reported at routine follow-up visits. Initiatives to develop more rigorous data collection methods and to develop composite pain outcomes combining patient-reported and clinical information collected throughout the study, not just at fixed intervals, are encouraged in clinical trials of degenerative spinal conditions. Such efforts may enhance physician and patient understanding of therapeutic efficacy and avoid misleading conclusions.

## Figures and Tables

**Table 1:** Unadjusted and integrated VAS pain scores with ACD and controls at each follow-up visit.

**Follow-up Interval**	**Unadjusted ** ^**a**^			**Integrated ** ^**b**^		
	**ACD**	**Control**	***p*-value**	**ACD**	**Control**	***p*-value**
Pre-treatment	81±15	81±15	0.97	81±15	81±15	0.97
6 weeks	13±19	19±25	0.001	14±21	22±29	<0.001
3 months	12±20	15±22	0.08	14±23	19±27	0.02
6 months	11±18	15±23	0.03	13±22	22±32	<0.001
1 year	11±19	14±21	0.13	16±27	26±34	<0.001
2 years	12±21	14±21	0.33	19±30	29±36	<0.001

**Notes:** ^a^values represent patient-reported VAS scores (mean±SD) recorded at scheduled follow-up visits; ^b^values represent adjusted VAS scores (mean±SD) where the baseline VAS pain score was substituted for patient-reported VAS score at the time of symptomatic reherniation and at each subsequent follow-up visit through 2 years using the BOCF approach to represent clinical failure. ACD, annular closure device; BOCF, baseline observation carried forward; SD, standard deviation; VAS, visual analogue scale.

**Table 2:** Unadjusted and integrated VAS pain nonresponders with ACD and controls at each follow-up visit.

**Follow-up Interval**	**Unadjusted ** ^**a**^			**Integrated ** ^**b**^		
	**ACD**	**Control**	***p*-value**	**ACD**	**Control**	***p*-value**
6 weeks	6.4%	10.7%	0.09	8.3%	15.6%	0.01
3 months	5.3%	6.8%	0.47	7.9%	12.0%	0.15
6 months	4.7%	5.7%	0.69	7.7%	16.6%	0.002
1 year	5.4%	7.7%	0.29	13.0%	25.1%	<0.001
2 years	6.0%	6.7%	0.89	15.7%	27.8%	0.001

**Notes:**  ^a^values represent percentage of patient-reported VAS nonresponders (improvement < 20 points from baseline) recorded at scheduled follow-up visits; ^b^values represent percentage of patient-reported VAS nonresponders (improvement < 20 points from baseline) where the baseline VAS pain score was substituted for patient-reported VAS score at the time of symptomatic reherniation and at each subsequent follow-up visit through 2 years using the BOCF approach to represent clinical failure. ACD, annular closure device; BOCF, baseline observation carried forward; VAS, visual analogue scale.

## Data Availability

The clinical trial data used to support the findings of this study have not been made available due to commercial confidentiality.
